# A case of iatrogenic immunodeficiency-associated colonic lymphoma complicating ulcerative colitis

**DOI:** 10.1186/s13000-020-00954-8

**Published:** 2020-04-07

**Authors:** Kazushi Suzuki, Rintaro Ohe, Takanobu Kabasawa, Naing Ye Aung, Mitsuhiro Yano, Shuichiro Katsumi, Ryo Yanagiya, Masakazu Yamamoto, Tomomi Toubai, Kenichi Ishizawa, Mitsunori Yamakawa

**Affiliations:** 1 Department of Pathological Diagnostics, Yamagata University Faculty of Medicine, 2-2-2 Iida-Nishi, Yamagata, 990-9585 Japan; 2 Department of Gastroenterological, General, Breast and Thyroid Surgery (First Department of Surgery), Yamagata University Faculty of Medicine, Yamagata, Japan; 3 Department of Gastroenterology, Sanyudo Hospital, Yonezawa, Japan; 4 Department of Neurology, Hematology, Metabolism, Endocrinology and Diabetology, Yamagata University Faculty of Medicine, Yamagata, Japan

## Abstract

**Background:**

Ulcerative colitis (UC) is one of the major types of inflammatory bowel diseases and is associated with a significantly increased risk of not only lymphoproliferative disorders but also lymphomas, of which most cases are related to the long-term usage of immunosuppressants. Here, we demonstrate a very rare case of other iatrogenic immunodeficiency-associated colonic diffuse large B-cell lymphoma (Oii-DLBCL) complicating UC and rectal perforation. In addition, we reviewed the clinicopathological features of previous cases of DLBCL related to UC.

**Case presentation:**

A 68-year-old man was diagnosed with left-sided UC 26 months prior. Although he was followed by immunosuppressive therapy with azathioprine and infliximab, an emergency total proctocolectomy was performed due to rectal perforation. The resected specimen exhibited irregular wall thickening and elevated multinodular lesions extending from the mid-transverse colon to the rectum, measuring up to 52 cm in length. Histologically, the lesion was diagnosed as Oii-DLBCL and crypt abscess surrounded by mixed inflammatory cell was remained.

**Conclusion:**

Oii-DLBCL complicating UC with rectal perforation is extremely rare. Macro- and microscopic findings are important for early diagnosis of the lesion.

## Background

It is generally accepted that inflammatory bowel disease (IBD) itself is not a risk factor for lymphomas [1]. Lymphomas complicating ulcerative colitis (UC), which are known as other iatrogenic immunodeficiency-associated lymphoproliferative disorders (Oii-LPDs), are caused by immunosuppressants such as azathioprine, 6-mercaptopurine, and infliximab [2, 3].

According to data found in PubMed between 2001 and 2019, only ten cases of diffuse large B-cell lymphoma (DLBCL) complicating UC, including one case of large B-cell lymphoma with unknown details, have been reported [1, 3–11]. Approximately half of these cases are closely related to the long-term usage of immunosuppressants (70%, 7/10 cases) and a very high rate of Epstein-Barr virus (EBV) infection (80%, 4/5 cases) and are classified as Oii-LPD. In Oii-LPD, an EBV-positive mucocutaneous ulcer (EBVMCU) is a specific type of immunosuppression-associated LPD due to the long-term usage of iatrogenic immunosuppressants or age-related immunosenescence [2, 12].

Here, we report a very rare case of Oii-LPD complicating UC and rectal perforation. At first, the histological findings were similar to those of DLBCL or EBVMCU. Then, the macroscopic and histological findings with the aid of immunophenotypic analysis suggested that the lesion was of the Oii-LPD, DLBCL phenotype.

## Case presentation

### Clinical presentation

A 68-year-old man was diagnosed with left-sided UC 26 months prior and was followed up without any treatment. He was first treated with 5-aminosalicylate because of worsening of diarrhea. A dose of 30–50 mg/day of prednisolone was also administered for the control of diarrhea and bloody stools. However, his symptoms repeatedly recurred with the sequentially decreasing usage of prednisolone. He was treated with an initial dose of 25 mg/day of azathioprine and a dose of 5 mg/kg twice daily of infliximab 8 months prior, but he could not obtain a satisfactory improvement in symptoms. Ultimately, he was diagnosed with pancolitic UC by colonoscopy. Ganciclovir was administered 1 month prior due to cytomegalovirus (CMV) antigenemia. Though the serum CMV antigen disappeared, the patient was transferred to our hospital with a complaint of a persistent fever and a perianal fistula. Immediately, abdominal computed tomography showed circumferential thickening of the rectal wall and a discontinuity in contrast enhancement of the posterior rectal wall. Because of suspicion of rectal perforation, an emergency total proctocolectomy was performed. After the total proctocolectomy, the patient achieved clinical remission without any additional treatment. There was no recurrence for 40 months after the operation, as shown by computed tomography. The resected specimen was fixed in 10% buffered formalin, and paraffin-embedded tissue sections were used for histological examination.

### Pathological findings

**Fig. 1:**
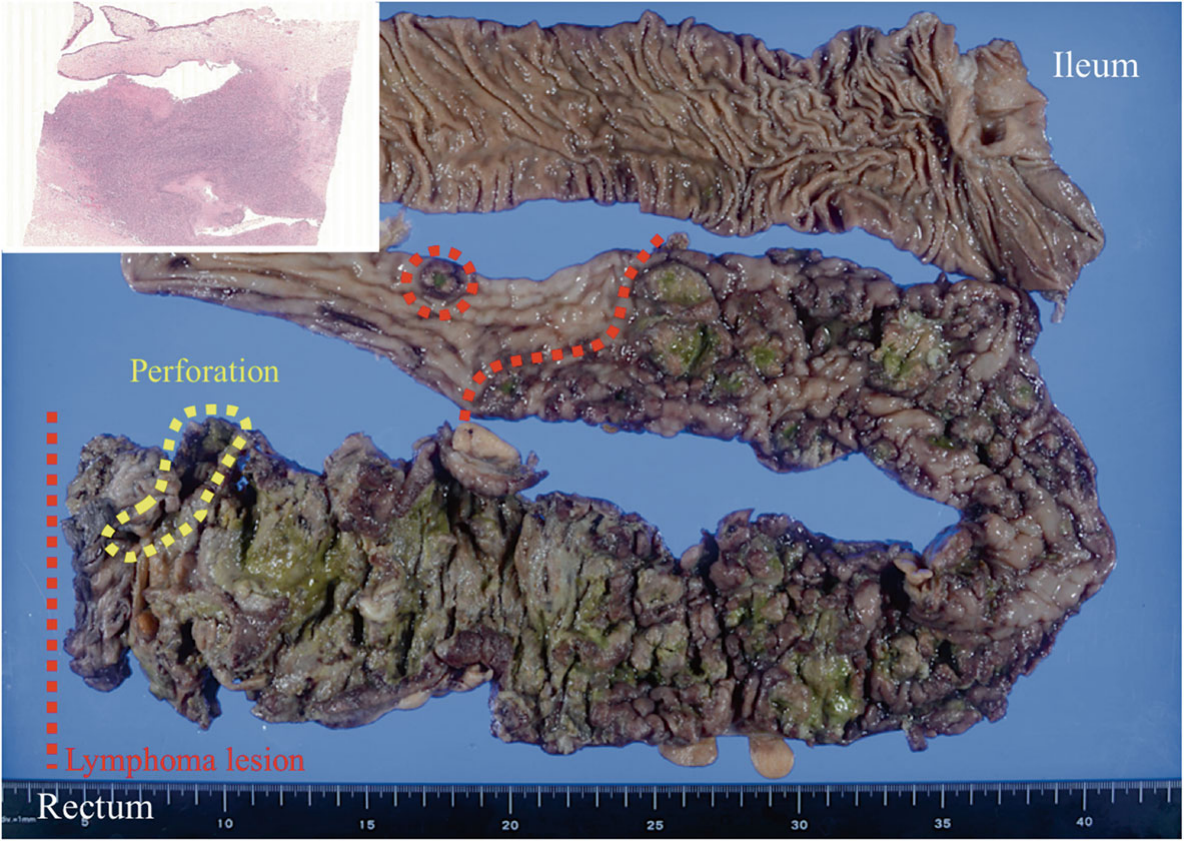
Total proctocolectomy specimen: Diffuse, irregular thickening of the colorectal wall extends from the middle of the transverse colon (*red broken lines*) to the rectum with a rectal perforation (*yellow circular broken line*). The image of perforation department is shown (*Inset*)

The resected specimen exhibited irregular wall thickening and elevated multinodular lesions extending from the mid-transverse colon to the rectum, measuring up to 52 cm in length (Fig. 1). Multiple sharply circumscribed ulcers and geographic necrosis were present. A rectal perforation, approximately 2 cm in diameter, was also noted. No abnormal mesenteric lymphadenopathy was noted.

**Fig. 2:**
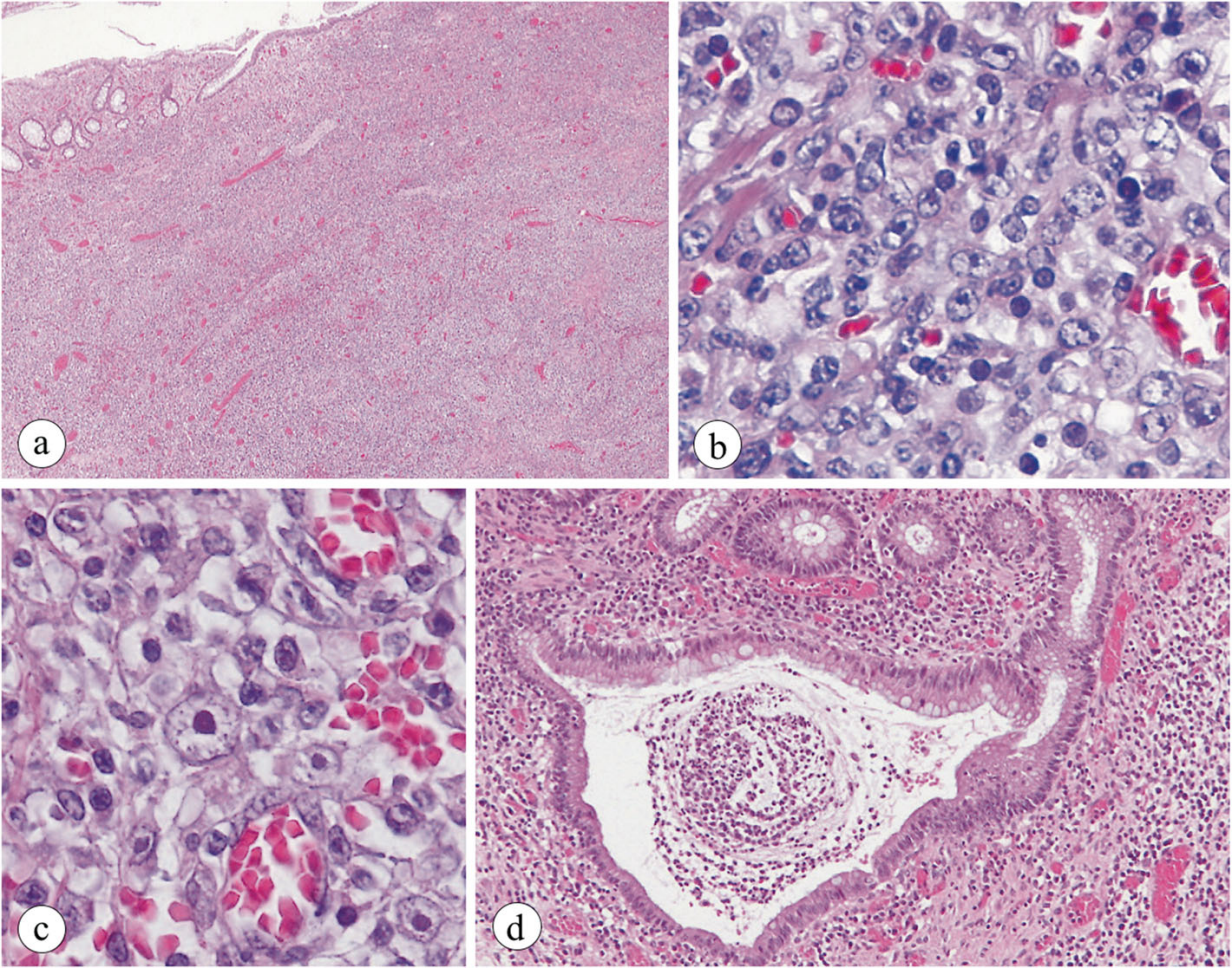
Histological findings of the resected colon: **a** The resected colon is transmurally infiltrated by lymphoid cells, and the existing structure is indistinct. **b** Atypical polymorphous cells diffusely proliferate. **c** A few Hodgkin-like cells with an eosinophilic nucleolus in the center of a large nucleus are present. **d** A crypt abscess surrounded by mixed inflammatory cell infiltrates is present in the lamina propria mucosae remote from the lymphoma, compatible with the findings of UC

**Fig. 3:**
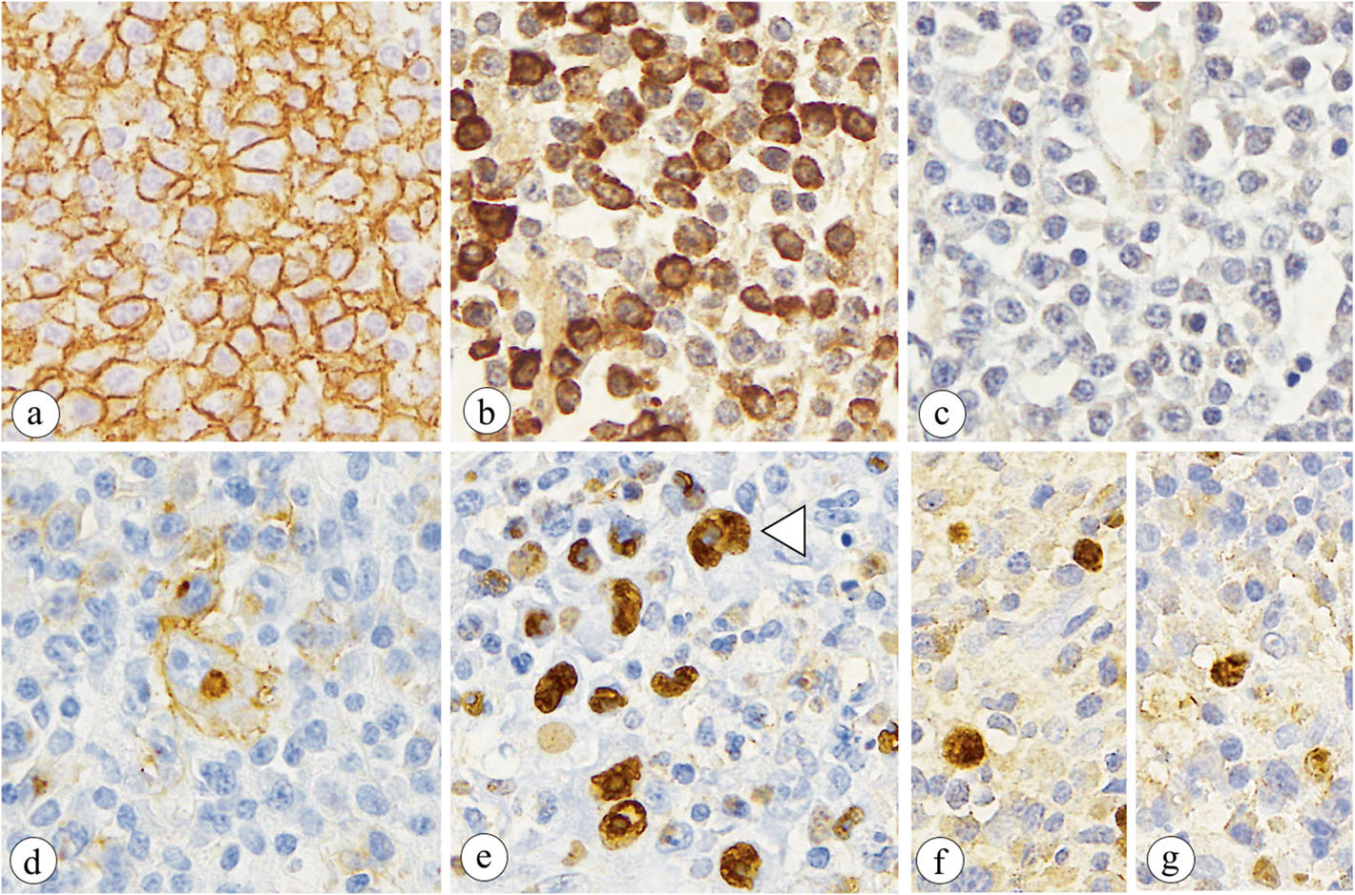
Immunophenotypic findings of the resected colon: Large, atypical polymorphous lymphoid cells are positive for CD20 by immunohistochemistry (**a**)**,** positive for the κ-light chain (**b**) and negative for λ-light chain (**c**) by in situ hybridization (ISH). Hodgkin-like cells are positive for CD30 by immunohistochemistry (**d**) and Epstein-Barr encoding region (EBER)-1 (**e**, **Arrowhead)** by ISH. Some large, atypical lymphoid cells also are positive for EBER-1 (**e**) by ISH. In a retrospective analysis, EBER-1-positive large atypical cells are present on the 9-day and 3-week colorectal tissue sections (**f** & **g**)

Microscopically, the resected lesion exhibited the diffuse transmural proliferation of lymphocytes and immunoblasts (Fig. 2a). Many polymorphic, large, atypical lymphocytes and scattered Hodgkin-like cells were present intermingled with scattered plasma cells, histiocytes and eosinophils in the background (Fig. 2b & c). Apoptotic bodies with plasmacytoid features were absent. Additionally, around the lymphoma, more remarkably in the distal colon than in the proximal colon remote to the lymphoma, admixed inflammatory cells composed of reactive lymphocytes with occasional lymphoid follicles, plasma cells including basal plasmacytosis, histiocytes, neutrophils often with cryptitis and crypt abscesses, and eosinophils infiltrated in the lamina propria mucosae with or without erosion or ulcers, indicating histological features compatible with UC, grade 5 in the maximal degree (Fig. 2d). Pleomorphic, large, atypical lymphocytes were immunohistochemically positive for CD20 (Fig. 3a), CD79a, MUM-1, and κ-light chain and negative for CD3, CD5, BCL-2, BCL-6, c-Myc, GCET1, Foxp1, and λ-light chain. The cells were also positive for κ-light chain but not λ-light chain according to in situ hybridization (ISH) (Fig. 3b & c). Scattered Hodgkin-like cells were immunohistochemically positive for CD20, CD30 (Fig. 3d), CD79a, PAX5, and LMP-1 (partially) but not CD10, CD15, BOB1, OCT2, BCL-6, or MUM-1. In both atypical lymphocytes and Hodgkin-like cells, Epstein-Barr encoding region (EBER)-1 positivity was detected by ISH (Fig. 3e). The base of the ulcer was rimmed by numerous medium-sized T-cells positive for CD3. Polymerase chain reaction (PCR) revealed a gene rearrangement of the immunoglobulin heavy chain but not the γ-chain of the T-cell receptor. Based on these clinical, histological and immunohistochemical findings, this lesion was diagnosed as Oii-LPD, DLBCL phenotype, polymorphous subtype. Large, atypical cells with a viral inclusion body were absent and immunohistochemically negative for CMV.

As a retrospective analysis, immunohistochemistry for CMV and ISH for EBER-1, κ-light chain, and λ-light chain were performed on paraffin-embedded tissue sections obtained from the colorectal biopsies of our patient at 9 days, 3 weeks, 6 months, 8 months, and 10 months before admission to our hospital. CMV-positive cells were present on the 3-week and 6-month tissue sections. EBER-1-positive large, atypical cells were present on the 9-day and 3-week tissue sections (Fig. 3f & g). There was no restriction of the κ-light chain or λ-light chain by ISH on any biopsied specimen.

## Discussion and conclusion

We reviewed the clinicopathological data of 11 cases of DLBCL complicating UC published in the English literature, including our case, as shown in Table 1 [1, 3–11]. The age at diagnosis ranged from 20 to 73 (median, 55) years. Surprisingly, this disease occurs more frequently in males than in females (male to female ratio = 10:1). The UC lesion was confined to the left side of the colon in 4 cases and extended to the whole colon in 5 cases. The duration of disease ranged from 26 to 300 (median, 84) months, and the duration of treatment with immunosuppressants ranged from 6 to 60 (median, 32) months. Both durations in our case were the shortest among all cases. Among these 6 available cases, 3 achieved remission and 3 died. Infliximab was used in 4 cases. However, it is difficult to conclude that treatment with infliximab is related to the increased risk of lymphoma because there are few cases of patients treated by infliximab monotherapy, and the influence of the azathioprine combination is unclear [1]. Conversely, there were 3 cases with DLBCL complicating UC without immunosuppressant treatment, although IBD itself is not a risk factor of lymphoma [1]. Surprisingly, colorectal perforation occurred in 4 cases, including ours. Our patient unavoidably underwent a proctocolectomy because of rectal perforation. The choice of surgical resection may not necessarily be better when the lesion is diagnosed as Oii-DLBCL. Therefore, a careful follow-up with recognition of the possibility of Oii-DLBCL complicating UC and early detection of the disease confirmed by an appropriate pathological diagnosis may be crucial to obtain a good prognosis.

**Table 1:** Clinicopathological data of diffuse large B-cell lymphoma (DLBCL) complicating ulcerative colitis published in English literature from 2001 to 2019

Authors, year	Age/Sex	Ulcerative colitis		Immunosuppressant treatment		Iatrogenic immunodeficiency-associated lymphoproliferative disorders					Outcome
		Site	Duration (month)	Immuno-suppressant	Duration (month)	Type	Site	Mass	EBV	perforation	
Tan, et al., 2001 [4]	36/M	Left-side	84	AZA	32	DLBCL	Rectum	+	N/A	N/A	Dead (due to renal insufficiency by the disease progression)
Khan, et al., 2001 [5]	55/M	Pancolic	300	None	None	LCL, B-cell lineage	Rectum	N/A	N/A	N/A	Remission
Watanabe, et al., 2003 [6]	42/F	Left-side	120	None	None	DLBCL	Colon	+	N/A	N/A	Dead (due to MRSA pneumonia after the transplant)
Schwartz, et al., 2006 [7]	29/M	Pancolic	48	6MP, IFX, CYA	24	DLBCL	Ileal pouch	+	+	N/A	Remission
Shibahara, et al., 2006 [8]	33/M	Pancolic	108	AZA, CYA	48	DLBCL	Rectum	+	N/A	N/A	Remission
Van Hauwaert, et al., 2010 [9]	20/M	N/A	N/A	AZA, IFX	60	DLBCL	Rectum	+	–	N/A	N/A
Khan, et al., 2012 [10]	59/M	N/A	132	None	None	DLBCL	Rectum	+	N/A	+	N/A
Allen, et al., 2013 [1]	65/M	Left-side	60	AZA, 6MP, IFX	60	DLBCL	Colon	+	+	+	N/A
Hiyama, et al., 2014 [11]	69/M	Left-side	42	AZA	24	DLBCL	Rectum	-; Ulcer	+	+	Dead (due to DIC secondary to sepsis)
Chang, et al. 2016 [3]	73/M	Pancolic	N/A	MTX	Several years	DLBCL	Colon	+	+	N/A	N/A
Our case	68/M	Pancolic	26	AZA, IFX	6	DLBCL	Colon	+	+	+	Remission

*M* male, *F* female, *AZA* azathioprine, *6MP* 6-mercaptopurine, *IFX* infliximab, *CYA* cyclosporine A, *MTX* methotrexate, *LCL* large cell lymphoma, *LN* lymph node, *EBV* Epstein-Barr virus, *N/A* not available, *MRSA* methicillin-resistant *Staphylococcus aureus*, *DIC* disseminated intravascular coagulation syndrome

EBVMCU is recognized by characteristic histological features, such as well-circumscribed ulcers and the infiltration of polymorphous cells (e.g., Hodgkin/Reed-Sternberg-like cells, numerous medium-sized T-cells, and apoptotic bodies with plasmacytoid features) in the background of lymphoma [13]. Natkunam et al. proposed additional findings, including ulcerative lesions without a mass, the rimming of small T-cells at the ulcer base, and detection of the clonal immunoglobulin or T-cell receptor gene rearrangement [14]. The lesions described in the present case were grossly composed of mostly circumscribed ulcers and multiple nodules without apparent voluminous mass lesions, measuring 52 cm in length. Histologically, large B-cells diffusely proliferated intermingled with scattered CD30-positive Hodgkin/Reed-Sternberg-like cells and numerous medium-sized CD3-positive T-cells rimming the base of the ulcer. However, plasmacytoid apoptotic bodies were absent. Clonal immunoglobulin κ-light chain was detected by immunohistochemistry and ISH. PCR also revealed a gene rearrangement of the immunoglobulin heavy chain but not the γ-chain of the T-cell receptor. Although these findings suggested EBVMCU, this patient was diagnosed with Oii-LPD, DLBCL phenotype.

Satou A et al. speculated that defective immune surveillance may be caused by a strong suppression of EBV-specific cytotoxic T-cell function, leading to CMV colitis [15], which is a candidate parameter of immunosuppression but not a causative event of the evolution of Oii-LPD. In our case, CMV infection was treated with ganciclovir, and CMV-positive cells were not detected immunohistochemically on either the surgical specimen or the colorectal biopsy specimen 9 days before admission or 3 days before admission to our hospital.

In conclusion, Oii-DLBCL complicating UC with rectal perforation is extremely rare and poorly understood. Macro- and microscopic findings suggested EBVMCU; these findings included well-circumscribed ulcers without an evident voluminous mass lesion, polymorphous infiltration, the absence of plasmacytoid apoptotic bodies, and the rimming of small T-cells at the ulcer base, as well as detection of the clonal immunoglobulin or T-cell receptor gene rearrangement, which are important for early diagnosis of the lesion. Furthermore, it should be emphasized that colonic perforation frequently occurs in cases of Oii-DLBCL.

## Data Availability

Is available upon request from the corresponding author.

## Abbreviations

IBD: Inflammatory bowel disease
UC: Ulcerative colitis
Oii-LPD: Other iatrogenic immunodeficiency-associated lymphoproliferative disorder
DLBCL: Diffuse large B-cell lymphoma
EBVMCU: EBV-positive mucocutaneous ulcer
CMV: Cytomegalovirus
ISH: In situ hybridization
EBER: Epstein-Barr encoding region
PCR: Polymerase chain reaction

## References

[1] Allen PB, Laing G, Connolly A, O'Neill C. EBV-associated colonic B-cell lymphoma following treatment with infliximab for IBD: a new problem? BMJ Case Rep. 2013;2013. 10.1136/bcr-2013-200423 PMC3794187 24081592

[2] Gaulard P, Swerdlow SH, Harris NL, Jaffe ES, Swerdlow SH, Campo E, Harris NL, C. S (2017). Other iatrogenic immunodeficiency-associated lymphoproliferative disorders. WHO classification of tumors of hematopoietic and lymphoid tissues. Revised 4th ed.

[3] Chang MD, Markham MJ, Liu X (2016). Epstein-Barr virus-positive diffuse large B-cell lymphoma involving the Colon in a patient with ulcerative Pancolitis and Polymyositis on long-term methotrexate therapy. Gastroenterol Res.

[4] Tan CW, Wilson GE, Howat JM, Shreeve DR (2001). Rectal lymphoma in ulcerative colitis treated with azathioprine. Eur J Gastroenterol Hepatol.

[5] Khan S, Anderson GK, Eppstein AC, Eggenberger JC, Margolin DA (2001). Ulcerative colitis and colonic lymphoma: a theoretical link. Am Surg.

[6] Watanabe N, Sugimoto N, Matsushita A (2003). Association of intestinal malignant lymphoma and ulcerative colitis. Intern Med.

[7] Schwartz LK, Kim MK, Coleman M, Lichtiger S, Chadburn A, Scherl E (2006). Case report: lymphoma arising in an ileal pouch anal anastomosis after immunomodulatory therapy for inflammatory bowel disease. Clin Gastroenterol Hepatol.

[8] Shibahara T, Miyazaki K, Sato D (2006). Rectal malignant lymphoma complicating ulcerative colitis treated with long-term cyclosporine a. J Gastroenterol Hepatol.

[9] Van Hauwaert V, Meers S, Verhoef G (2010). Rectal non-Hodgkin's lymphoma in an infliximab treated patient with ulcerative colitis and primary sclerosing cholangitis. J Crohns Colitis.

[10] Khan A, Lloyd GM, Ihedioha U, Hemingway D (2012). Rectovesical fistula secondary to B-cell lymphoma of the rectum: a unique presentation of a rare disease. Color Dis.

[11] Hiyama K, Terashima H, Nakano Y (2015). Primary rectal diffuse large B-cell lymphoma associated with ulcerative colitis: a case report. Clin Case Rep.

[12] Gaulard P, Swerdlow SH, Harris NL, Jaffe ES, Swerdlow SH, Campo E, Harris NL, C. S (2017). EBV-positive mucocutaneous ulcer. WHO classification of tumors of hematopoietic and lymphoid tissues. Revised 4th ed.

[13] Dojcinov SD, Venkataraman G, Raffeld M, Pittaluga S, Jaffe ES (2010). EBV positive mucocutaneous ulcer--a study of 26 cases associated with various sources of immunosuppression. Am J Surg Pathol.

[14] Natkunam Y, Goodlad JR, Chadburn A (2017). EBV-positive B-cell proliferations of varied malignant potential: 2015 SH/EAHP workshop report-part 1. Am J Clin Pathol.

[15] Satou A, Kohno A, Fukuyama R, Elsayed AA, Nakamura S (2017). Epstein-Barr virus-positive mucocutaneous ulcer arising in a post-hematopoietic cell transplant patient followed by polymorphic posttransplant lymphoproliferative disorder and cytomegalovirus colitis. Hum Pathol.

